# Path integral approach to the quantum fidelity amplitude

**DOI:** 10.1098/rsta.2015.0164

**Published:** 2016-06-13

**Authors:** Jiří Vaníček, Doron Cohen

**Affiliations:** 1 Laboratory of Theoretical Physical Chemistry, Institut des Sciences et Ingénierie Chimiques, Ecole Polytechnique Fédérale de Lausanne (EPFL), 1015 Lausanne, Switzerland; 2 Department of Physics, Ben-Gurion University of the Negev, Beer-Sheva 84105, Israel

**Keywords:** Loschmidt echo, quantum fidelity, dephasing representation, path integral

## Abstract

The Loschmidt echo is a measure of quantum irreversibility and is determined by the fidelity amplitude of an imperfect time-reversal protocol. Fidelity amplitude plays an important role both in the foundations of quantum mechanics and in its applications, such as time-resolved electronic spectroscopy. We derive an exact path integral formula for the fidelity amplitude and use it to obtain a series of increasingly accurate semiclassical approximations by truncating an exact expansion of the path integral exponent. While the zeroth-order expansion results in a remarkably simple, yet non-trivial approximation for the fidelity amplitude, the first-order expansion yields an alternative derivation of the so-called ‘dephasing representation,’ circumventing the use of a semiclassical propagator as in the original derivation. We also obtain an approximate expression for fidelity based on the second-order expansion, which resolves several shortcomings of the dephasing representation. The rigorous derivation from the path integral permits the identification of sufficient conditions under which various approximations obtained become exact.

## 1. Introduction

Because of the unitarity of quantum evolution, the overlap of two different quantum states remains constant in time. As a consequence, to measure the stability of quantum dynamics, one has to perturb the Hamiltonian rather than the initial state. For this purpose, Peres has introduced [1] the notion of *quantum fidelity*, defined for pure initial states *ψ* as *F*(*t*):=|*f*(*t*)|^2^, where
1.1
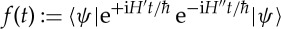
is the fidelity amplitude, *H*′ is the unperturbed Hamiltonian and *H*^′′^=*H*′+Δ*H* is the perturbed Hamiltonian. Equation (1.1) states that the fidelity amplitude is the overlap at time *t* of two identical initial states evolved with two different time-independent Hamiltonians.

Fidelity is also referred to as the *Loschmidt echo* [2] because it can be interpreted as the survival probability of an initial state *ψ* evolved for time *t* with Hamiltonian *H*′ and subsequently for time −*t* with *H*^′′^. It has been studied extensively in the past 15 years [3–5], leading to the identification of various universal regimes of its decay in time, which are closely related to similar observations in the theory of wavepacket dynamics and to the parametric regimes of the local density of states [6,7].

Quantum fidelity has a fundamental role in our understanding of quantum irreversibility [8]; it provides another perspective to the theories of decoherence; and it is important for experimental realizations of quantum computation [9]. While several nuclear magnetic resonance [10,11], microwave [12] and atom optics [13,14] experiments were designed specifically to study the Loschmidt echo or fidelity amplitude, the same correlation function occurs naturally in linear and nonlinear electronic spectroscopy. For example, within the time-dependent perturbation theory and Condon approximation, electronic absorption or emission spectra, and time-resolved spectra, in particular, can be computed via a Fourier transform of an appropriately defined fidelity amplitude [15–17].

The Loschmidt echo has been studied by many different approaches, which are reviewed in [3–5]. Here we focus on a path integral approach, in order to gain further understanding of the often used semiclassical methods. Indeed, many of the analytical expressions for fidelity decay were obtained by the original semiclassical approach of Jalabert & Pastawski [18], while Cerruti & Tomsovic [19] performed the first numerical semiclassical calculation, in which they found explicitly approximately 1000 stationary-phase contributions to the fidelity amplitude. Vaníček & Heller [20] avoided the search for stationary-phase points and obtained a uniform expression for fidelity by combining Miller’s initial value representation [21,22] with the semiclassical perturbation approximation [23]. This surprisingly simple and accurate expression, although limited to wave packets localized in position, was successfully applied as a starting point to derive the decay of fidelity in the deep Lyapunov regime [24] and the plateau of fidelity in neutron scattering [25]. By linearizing the semiclassical initial value representation of the fidelity amplitude, Vaníček later obtained [26,27] a more general and accurate approximation, the so-called *dephasing representation*,
1.2
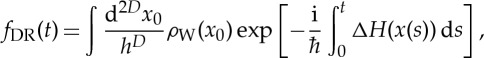
applicable not only to pure states (*ρ*=|*ψ*〉〈*ψ*|), but also to arbitrary mixed initial states *ρ*. In equation (1.2), *D* is the number of degrees of freedom, *x*:=(*q*,*p*) is a collective notation for positions *q* and momenta *p*, 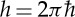 is the Planck constant, *x*(*t*) denotes the phase-space coordinates at time *t* of a trajectory of the average Hamiltonian *H*:=(*H*′+*H*^′′^)/2 with initial condition *x*_0_, and *ρ*_W_ is the Wigner function, i.e. the Wigner transform of the density operator *ρ* of the initial state. Note that we use the following convention for the Wigner transform of a general operator *A*:

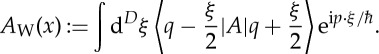


In electronic spectroscopy, the dephasing representation and closely related approximations are known as Mukamel’s phase-averaging method [15,28] or the Wigner-averaged classical limit, and have been used by various authors [29–32]. In the context of the mixed quantum–classical Liouville equation, Martens and co-workers obtained a similar expression for the evolution of coherences of the density operator [33,34]. In the field of quantum chaos, the dephasing representation successfully described, for example, the local density of states and the transition from the Fermi’s golden rule to the Lyapunov regime of fidelity decay [35–37].

Yet the most attractive feature of the dephasing representation is its efficiency: motivated by numerical comparisons with other semiclassical methods [16], it was proved analytically [38] that the number of trajectories required for convergence of the dephasing representation was independent of the system’s dimensionality, Hamiltonian, or total evolution time. Unlike its efficiency, the accuracy of the dephasing representation is not always sufficient. This approximation is exact in displaced harmonic oscillators [15,28] and often accurate in chaotic systems [26,27], but it breaks down in such simple systems as harmonic oscillators with different force constants. This problem can be partially remedied by augmenting the approximation with a prefactor [39,40], which, however, is still not exact even for harmonic systems.

*Outline*. The present paper was motivated by two goals: first, to derive the dephasing representation from the Feynman path integral, without employing the semiclassical propagator; and, second, to obtain a semiclassical approximation correcting the drawbacks of the original version of the dephasing representation. Below, we do exactly that, but on the way also obtain a recipe for obtaining increasingly accurate semiclassical approximations from the expansion of the path integral, and explicit expressions for the zeroth-, first- and second-order expansions. As we will see, the first-order expansion yields the original dephasing representation, and its inaccuracies can be corrected with the second-order expansion. The paper is organized as follows. First, in §2, we derive the coordinate-space path integral representation of the fidelity amplitude by analogy with the path integral for the classical Liouville propagator and quantum propagator of the density operator. Then, in §3, we provide an alternative and more explicit phase-space path integral representation of the fidelity amplitude in kicked quantum maps, which allows us to obtain the zeroth-, first- and second-order approximations. Section 4 discusses under which circumstances various approximations are exact, while §5 concludes the paper.

## 2. Coordinate-space path integral representation

In order to simplify our first derivation of a path integral representation of *f*(*t*), in this section, we will consider one-dimensional systems described by the Hamiltonian
2.1
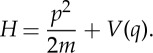
The derivation is based on analogies with path integral propagators of classical and quantum densities, which were discussed in detail by Cohen [41] for systems with noise.

### (a) Quantum propagator

The quantum propagator of a wave function can be obtained from the well-known Feynman path integral expression
2.2
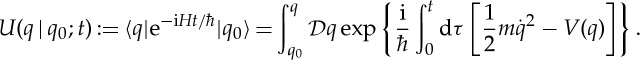
The density operator evolves as 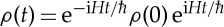; accordingly, its temporal evolution can be expressed by a propagator 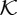 as
2.3
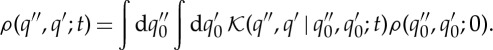
The propagator 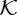 of the density operator is trivially related to *U*, namely,
2.4
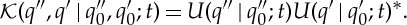
Consequently, the path integral expression for 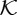 involves summation 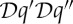 over the pair of paths *q*′(*τ*) and *q*^′′^(*τ*). Alternatively, we may also use the average and difference coordinates *q*:=(*q*′+*q*^′′^)/2 and *r*:=*q*^′′^−*q*′; thus the summation will be 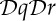, namely
2.5
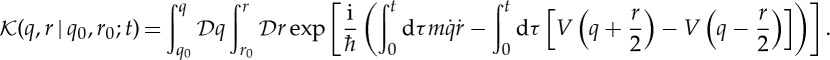
As a final step, we transform the quantum propagator to the Wigner representation. Recall that *ρ*_W_(*q*,*p*) is the Fourier transform of *ρ*(*q*,*r*) in the *r*↦*p* coordinate. It follows that
2.6
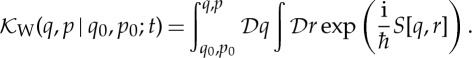
The integration 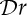 in the latter expression is not restricted at the endpoints, whereas the integration 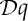 is restricted at the endpoints in both *q* and 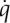. The restriction on 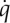 at the endpoints is implicit, through the relation 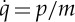. We have used the notation
2.7
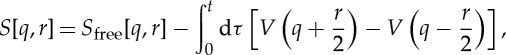
where
2.8
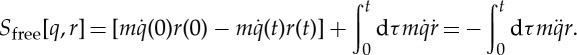
In the next subsection, we clarify that the leading-order estimate of the quantum propagator leads to the expected classical result.

### (b) Classical propagator

The time evolution of a classical phase-space density *ρ*_cl_(*q*,*p*;*t*), under the dynamics that is generated by a classical Hamiltonian (2.1), is given by the so-called Liouville propagator. For an infinitesimal time d*τ*, the explicit expression for the Liouville propagator is
2.9
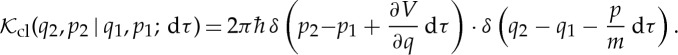
Here a dummy parameter 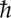 has been inserted, which cancels with the phase-space measure 
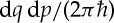
. Its value does not have any effect here, but the use of 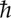 will make a later comparison with the quantum mechanical version more transparent. The inverse Fourier-transformed (*p*↦*r*) version, 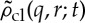, of phase-space representation *ρ*_cl_(*q*,*p*;*t*) is analogous to the coordinate-space representation *ρ*(*q*,*r*;*t*) of the quantum density matrix. (A tilde will be used on classical densities and propagators in the coordinate representation, i.e. if their arguments are *q* and *r*, or *q*′ and *q*^′′^.) The associated Fourier-transformed version of the classical Liouville propagator is accordingly
2.10
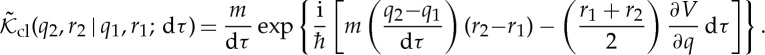
For a finite time, the convolved propagator may be written as a functional integral,
2.11
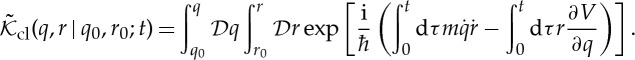
Transforming back to the phase-space variables, we get
2.12
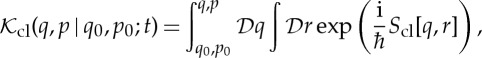
where the classical action is
2.13
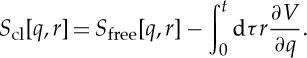
Note that the classical action is the same as the leading-order *r* expansion of the quantum action (2.7).

### (c) Fidelity amplitude

Now we use the same procedure to obtain an expression for the quantum fidelity amplitude at time *t* assuming that the initial preparation is described by the density matrix *ρ*(*q*^′′^,*q*′), and the two Hamiltonians differ only in their potential energies *V* ′(*q*) and *V* ^′′^(*q*). The following is the exact Feynman path integral with unrestricted integration over all possible paths:
2.14
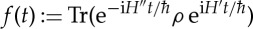

2.15
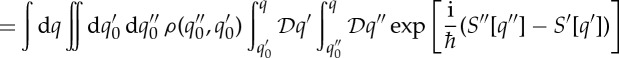

2.16
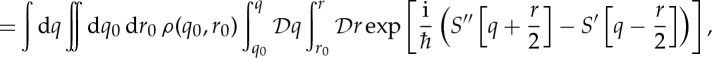
where the single-primed quantities such as *S*′ correspond to the evolution with *H*′ and the double-primed quantities such as *S*^′′^ correspond to *H*^′′^. We now use exactly the same manipulations as in §2a and write this expression using phase-space variables:
2.17
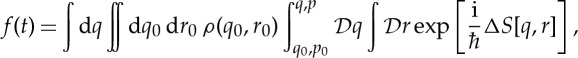
where
2.18
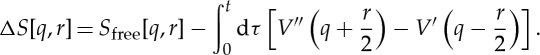
This expression is in one-to-one correspondence with (2.7); so far, no approximations were involved. The next step is to expand in *r*, namely
2.19
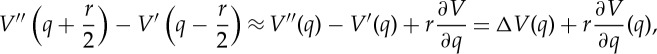
where *V* :=(*V* ′+*V* ^′′^)/2. Recall that, in the calculation of the quantum propagator, this linear approximation merely led to the classical propagator as Δ*V* (*q*) was zero. Here we shall see that the linearization leads to non-trivial quantum results. Note that the approximated action, including the ‘free’ action of (2.8), is linear in the *r*(*τ*) variables. Also it is possible to express *ρ*(*q*_0_,*r*_0_) as a Fourier integral over *ρ*_W_(*q*_0_,*p*_0_), involving 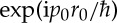. So now all the *r*(*τ*) including *r*_0_ appear in a linear fashion in the exponent. Consequently, the unrestricted 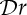 integration, including the d*r*_0_ integration, results in a product of delta functions. Subsequently, the 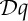 integration, including the final d*q* integration, picks up only the classical trajectories *q*_cl_(*τ*). We are left with the following very simple approximation:
2.20
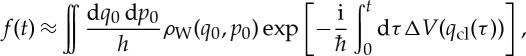
which coincides with the dephasing representation (1.2).

## 3. Phase-space path integral representation

In this section, we will use a phase-space path integral approach and generalize the analysis of the previous section by considering a system with *D* degrees of freedom described by the separable Hamiltonian
3.1
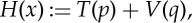
where *T*(*p*) and *V* (*q*) are arbitrary functions describing the kinetic and potential energies.

### (a) Quantum propagator

For short times *τ*, the quantum evolution operator 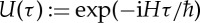 corresponding to Hamiltonian *H* can be approximated as
3.2
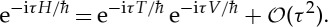
In order to avoid questions of convergence of the path integral and to make our derivations rigorously exact for as long as possible, we will consider kicked quantum maps, in which the error term in the factorization (3.2) is zero by definition. In other words, in a kicked quantum map, the evolution operator for a single time step is *defined* to be
3.3
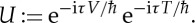


The quantum propagator from position *q*_*n*_ to *q*_*n*+1_ in a single time step of the map,
3.4
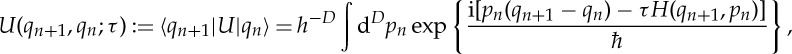
is obtained by inserting the resolution of identity 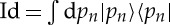 between the potential and kinetic evolution operators in (3.3). By concatenating *N* single-step propagators, one finds the propagator from *q*_0_ to *q*_*N*_ in time *t*=*Nτ*:
3.5
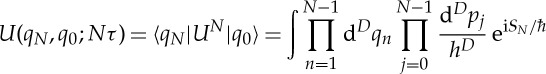
and
3.6
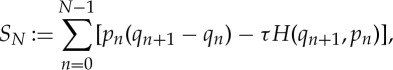
where *q*_*n*_ and *p*_*n*_ denote the positions and momenta after *n* steps. An appealing feature of the phase-space path integral is the absence of a complicated prefactor; one only has to consistently use the standard phase-space measure d^*D*^*q* d^*D*^*p*/*h*^*D*^.

### (b) Fidelity amplitude

To find the path integral representation of fidelity amplitude (1.1), we first express *f*(*t*) in terms of the quantum propagators:
3.7
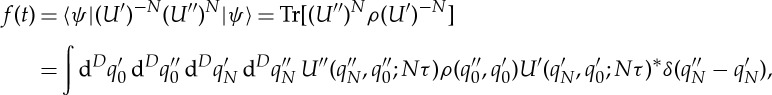
where the single-primed quantities such as *U*′ again correspond to *H*′ and double-primed quantities such as *U*^′′^ to *H*^′′^. By having expressed fidelity amplitude as a trace of the evolved density *ρ*, all our derivations below remain valid for general mixed states. After substituting the path integral expression (3.5) for the two propagators, we get
3.8
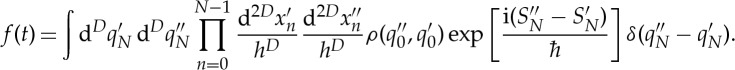
Now it is convenient to change the independent integration variables to the average and difference coordinates *x*:=(*x*′+*x*^′′^)/2 and Δ*x*:=*x*^′′^−*x*′,
3.9
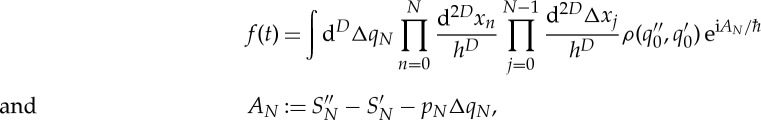
where we have also expressed the delta function *δ*(Δ*q*_*N*_) in terms of an integral over a new variable *p*_*N*_. After substituting the *N*-step action (3.6) for 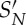 and 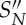 and simplification, one obtains an explicit expression for the phase,
3.10
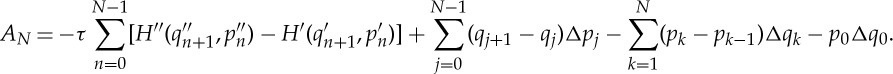
Note that expression (3.9) with (3.10) is *exact* for kicked quantum maps even for finite *N*.

### (c) Expansion of the path integral

The explicit expressions above in terms of the average and difference trajectories *x*_*n*_ and Δ*x*_*n*_ will now pay off because we can make increasingly more accurate expansions of the difference *H*^′′^(*x*^′′^)−*H*′(*x*′) in powers of Δ*x*, which is the only term in the exponent *A*_*N*_ preventing us from performing the path integral (3.9) analytically. This expansion must be done with care because *both* the trajectory and Hamiltonian change. Let us start with the full expansion, which is guaranteed to be exact if both *H*′ and *H*^′′^ have Taylor series that converge on the entire phase space:
3.11
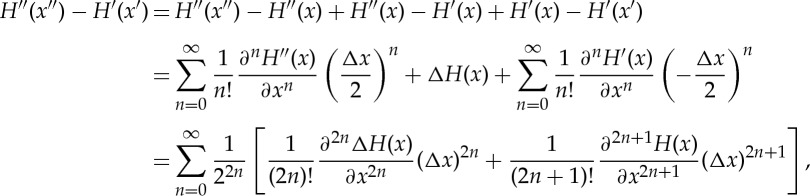
where we have introduced the *average HamiltonianH*:=(*H*′+*H*^′′^)/2 and the difference Hamiltonian (or *perturbation*) Δ*H*:=*H*^′′^−*H*′. Note that, for simplicity, we have for the moment used one-dimensional notation, and moreover, as both *H*′ and *H*^′′^ are separable in coordinates and momenta, so are *H* and Δ*H*, and expressions such as (∂^*n*^*H*(*x*)/∂*x*^*n*^)(Δ*x*)^*n*^ stand for (∂^*n*^*T*(*p*)/∂*p*^*n*^)(Δ*p*)^*n*^+(∂^*n*^*V* (*q*)/∂*q*^*n*^)(Δ*q*)^*n*^, etc. There are two important observations to make.

First, in the Δ*x* expansion (3.11), derivatives of the average Hamiltonian *H* appear only with *odd* powers of Δ*x* and derivatives of the perturbation Δ*H* appear only with *even* powers of Δ*x*. Second, the average Hamiltonian appears naturally and plays a prominent role. The average Hamiltonian must be used in order to preserve the order of the expansion. Otherwise (e.g. if *H*′ were used as a reference in displaced harmonic oscillators) what appears to be a first-order expansion in Δ*x* would in fact be of second order. This has a consequence, explained below in §4, that in displaced harmonic oscillators, the dephasing representation (1.2) mentioned in the introduction is exact if the average Hamiltonian *H* is used as reference, but not if *H*′ is used instead (see equation (4.3)).

It turns out to be useful to truncate expansion (3.11) at increasing powers of Δ*x*. As we will see later, both the zeroth- and first-order expansions yield simple analytical results, the latter agreeing exactly with the dephasing representation. The second-order expansion cannot be solved fully analytically, but nevertheless yields an appealing extension of the dephasing representation.

### (d) Zeroth-order expansion

Truncating expansion (3.11) at the zeroth power of Δ*x*, i.e. setting
3.12
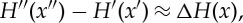
permits an analytical evaluation of almost all integrals in equation (3.9) as they involve either exponentials or delta functions. The result is the zeroth-order approximation of fidelity amplitude,
3.13
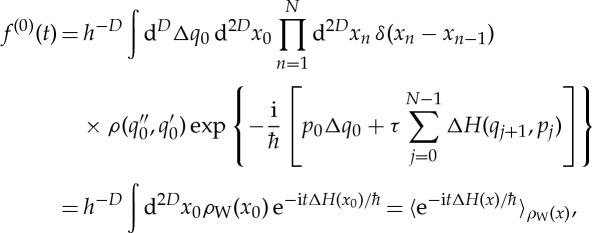
where *t*:=*Nτ* and the last expression employs the notation
3.14
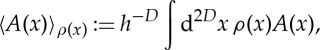
for a phase-space ‘average’ of a quantity *A* weighted by a normalized quasi-probability distribution *ρ*. Normalization means that 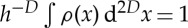, which is true for the Wigner function *ρ*_W_.

Note that, in equation (3.13), we have obtained a new approximation for quantum fidelity amplitude—one that is cruder than the dephasing representation (1.2) and does not even require running trajectories!

Although approximation *f*^(0)^ only requires phase-space sampling of the perturbation at initial time, in general, it yields a time-dependent fidelity amplitude. If one replaces *ρ*_W_ by the classical Boltzmann distribution, the zeroth-order approximation for fidelity amplitude coincides with an approximation used for calculations of inhomogeneously broadened spectra and known as the static classical limit [31,32].

*Example*: A sufficient condition for the zeroth-order approximation (3.13) for fidelity amplitude to be exact is that the zeroth-order expansion (3.12) itself is exact, which requires the average and difference Hamiltonians to be of the form *H*=*α* and Δ*H*=Δ*α*+Δ*β*⋅*q*+Δ*γ*⋅*p*, where *α*′, *α*^′′^, Δ*β* and Δ*γ* are constants, implying that the original Hamiltonians must be 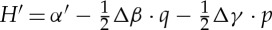 and 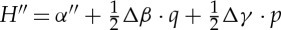. Corresponding classical motions are linear growth (or decrease) with time of phase-space coordinates for *H*′, *H*^′′^, and no motion at all for the average Hamiltonian *H*. Under such conditions, the zeroth-order approximation *f*^(0)^(*t*) is exact for arbitrary initial states *ρ*.

This can be verified independently by first expressing the fidelity amplitude as
3.15
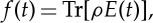
in terms of the echo operator
3.16
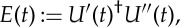
then using the phase-space representation of the trace in equation (3.15),
3.17
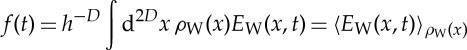
and finally evaluating explicitly the Wigner transform of the echo operator (3.16), which, after some algebra, in this case turns out to be 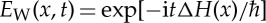, in agreement with equation (3.13).

Incidentally, the above sufficient condition is not necessary. For example, for Δ*H*=0, expression (3.13) is trivially exact, *f*^(0)^(*t*)=1, for arbitrary *H* even though one neglects the non-vanishing higher-order terms of the average Hamiltonian *H* in expansion (3.11).

### (e) First-order expansion

The first-order expansion of (3.11) approximates the Hamiltonian difference as
3.18
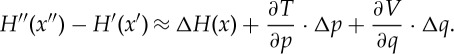
Again, most of the integrals can be solved analytically and one obtains, without any other approximation,
3.19
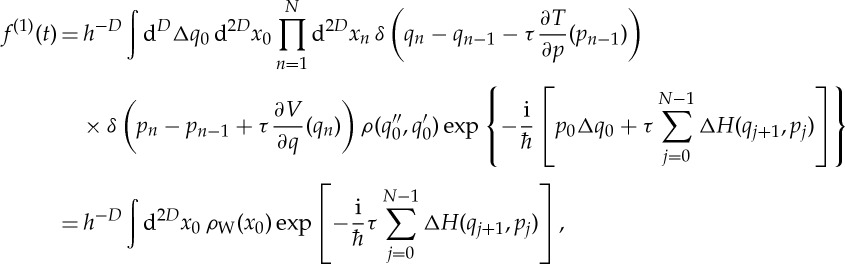
where *q*_*n*_ and *p*_*n*_ appearing as arguments of Δ*H* in the last expression are no longer independent path integral variables; instead, they are the uniquely defined position and momentum coordinates of a trajectory starting at *x*_0_ after *n* steps of the classical symplectic map given by the average Hamiltonian *H* and corresponding to the quantum map (3.3); these trajectories are given by the recursive relations between *q*_*n*_, *p*_*n*_ and *q*_*n*−1_, *p*_*n*−1_ expressed by the delta functions in the preceding equation.

To return from quantum maps to continuous Hamiltonian systems, one takes the limits 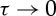 and 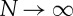, so that *Nτ*=*t* is constant, obtaining
3.20
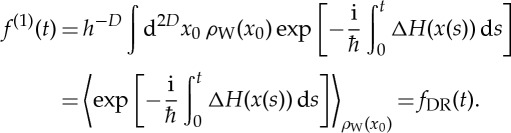
As promised, by using the first-order expansion of *H*^′′^−*H*′ in the path integral representation of quantum fidelity, we have obtained exactly the dephasing representation (1.2). On the one hand, this may seem remarkable, as we did not explicitly employ the semiclassical propagator which had been used in the original derivation of the dephasing representation [27]. On the other hand, the semiclassical propagator can be obtained by a quadratic expansion of the Feynman path integral propagator, and because we used a linearization of the path integral, we implicitly went beyond the semiclassical approximation since, in contrast with usual semi-classical approximations, expression (3.20) for *f*^(1)^≡*f*_DR_ does not even require Hessians of *H* or Δ*H*. Finally, we note that our result also agrees with a linearized path integral approximation obtained for a more general correlation function 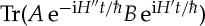 by a similar approach by Shi & Geva [42] in the context of non-radiative electronic relaxation rates.

*Example*: A sufficient condition for the first-order approximation (3.20) for fidelity amplitude to be exact is that the first-order expansion (3.12) itself is exact, which requires the average Hamiltonian to be at most a quadratic function, and the perturbation at most a linear function of positions and momenta, i.e.
3.21
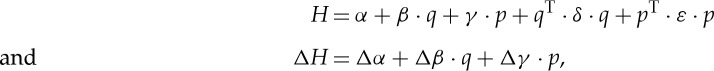
implying that the original Hamiltonians must be of the form
3.22
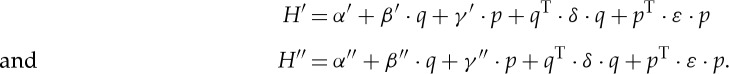
In other words, the two Hamiltonians describe harmonic (or inverted harmonic) systems that can be displaced in phase space, have different zeros of energy, but must have the same ‘masses’ and force constants in corresponding degrees of freedom. In one dimension, classical motions corresponding to Hamiltonians *H*′, *H*^′′^ are motions along ellipses or hyperbolas in phase space, where the centres of these conical sections in phase space may be displaced between *H*′ and *H*^′′^, but otherwise the phase portraits must be the same for the two Hamiltonians. For systems described by Hamiltonians (3.22), the first-order approximation *f*^(1)^(*t*), i.e. the dephasing representation, is exact for arbitrary initial states *ρ*. Such systems can be used to describe, for example, electronic absorption and emission spectra in molecules, where the displacement occurs only in coordinate space (i.e. Δ*β*≠0 and Δ*γ*=0) and results in vibrational excitation of a molecule upon electronic absorption. By contrast, Hamiltonians with displacement in momentum space (Δ*β*=0 and Δ*γ*≠0) are useful for representing inelastic collisions, such as inelastic neutron scattering [25].

Indeed, it is not surprising that the first-order approximation (3.20) is exact for quadratic Hamiltonians with linear perturbation, as many semiclassical approximations are exact in such situations. What is intriguing about the dephasing representation (3.20) is its surprising accuracy in chaotic systems. So the approximation is exact for Hamiltonians (3.22) and accurate in chaotic Hamiltonians, yet the most severe breakdown for it occurs in simple systems, such as quadratic Hamiltonians with quadratic perturbations. Next, we turn to deriving an expression that will correct this drawback.

### (f) Second-order expansion

In order to simplify the presentation of the second-order expansion, we shall assume that *D*=1 and Δ*H*(*x*)≡Δ*V* (*q*). The quadratic expansion of (3.11) approximates the Hamiltonian difference as
3.23
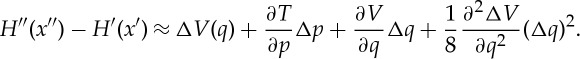
With this expansion, the phase (3.10) in the path integral representation (3.9) becomes
3.24
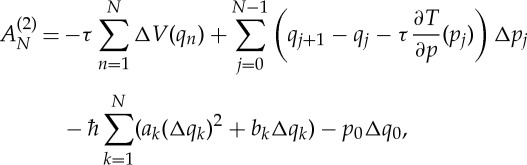
where
3.25
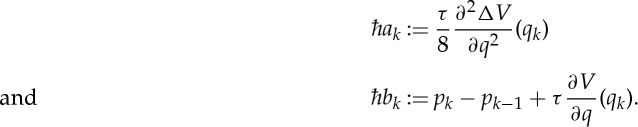
Again, the integrals over Δ*p*_*n*_ in (3.9) yield delta functions with arguments agreeing with Hamilton’s equations of motion for *q*_*n*_, and the integral over Δ*q*_0_ gives the Wigner function of the initial state:
3.26
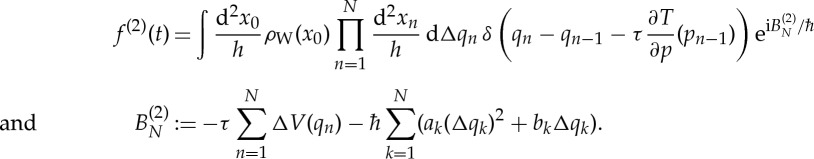
Although the complex Gaussian integrals over Δ*q*_*n*_ do not yield simple Dirac delta functions, they can be evaluated analytically and the fidelity amplitude becomes
3.27
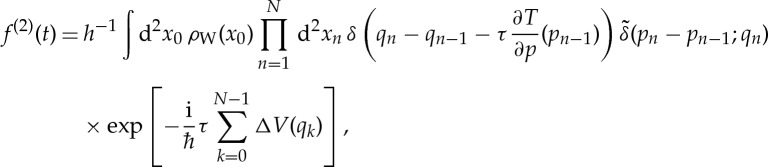
where 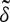 is a ‘smeared’ delta function, given by a complex Gaussian
3.28
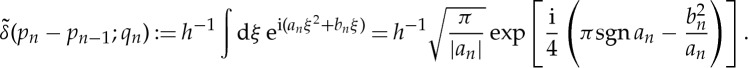
This smeared delta function replaces Hamilton’s equation for *p*_*n*_ with a ‘smeared Hamilton’s equation’—the expectation value of momentum *p*_*n*_ is still at the classical value *p*_*n*−1_−*τ*(∂*V* /∂*q*)(*q*_*n*_), but it is not deterministic as in classical mechanics. Equation (3.27) for the second-order fidelity amplitude thus has a simple interpretation, not unlike the dephasing representation: first, one samples initial conditions *x*_0_ from the density *ρ*_W_(*x*_0_). Then one runs trajectories starting from these points, where the kinetic propagation of positions is classical and hence deterministic, whereas the propagation of momenta is non-classical and stochastic. Although we have been able to evaluate three quarters of the integrals in the exact path integral representation (3.9) of *f*(*t*), the remaining *N* integrals over *p*_*n*_ render the resulting expression (3.27) still a formidable path integral, which is difficult to evaluate numerically. Note that, if we allowed the perturbation to affect also the momenta, then the propagation of positions would also be stochastic; the corresponding generalization of equation (3.27) is straightforward.

*Example*: A sufficient condition for the second-order approximation (3.27) for fidelity amplitude to be exact is that the second-order expansion (3.23) itself be exact, which requires the average Hamiltonian to be at most a quadratic function of *q* and *p*, and the perturbation at most a cubic function of *q*, i.e.
3.29
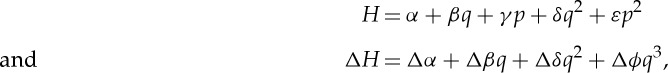
implying that the original Hamiltonians must be of the form
3.30
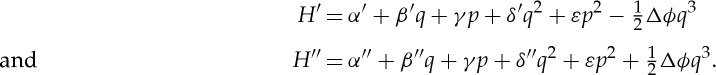


## 4. Discussion

The derivations based on the Feynman path integral bypass the conventional semiclassical approximations and therefore allow us to introduce several rigorous statements. If the Hamiltonian is up to *quadratic* and the perturbation up to *linear*, the dephasing representation (or phase averaging [15] or weighted average classical limit [31]) is exact. For example, for displaced simple harmonic oscillators
4.1
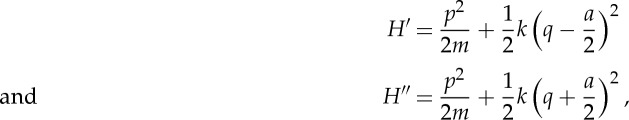
the dephasing representation is exact [15] if the classical trajectories are propagated with the average Hamiltonian *H* since then the Hamiltonian difference (3.11) is indeed linear in Δ*q* and Δ*p*:
4.2
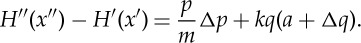
By contrast, the dephasing representation is *not* exact even in this simple system if *H*′ is used for dynamics, as quadratic terms in both Δ*q* and Δ*p* appear:
4.3
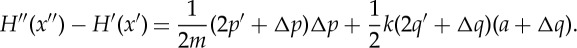
Similarly, the dephasing representation is *not* exact (in fact, breaks down rather severely) for simple harmonic oscillators with different force constants,
4.4
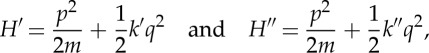
as the perturbation is quadratic in Δ*q* even if the average Hamiltonian is used for dynamics:
4.5
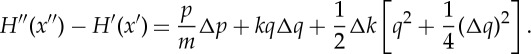
The last simple example provides a particularly bad scenario for the dephasing representation, which can be remarkably accurate in much more complex, even chaotic systems such as the kicked rotor. Unfortunately, undisplaced harmonic oscillators provide a good model for the ‘silent’ modes in electronic spectra, i.e. the modes which are not excited by the electronic transition, and hence are not displaced, but may have a different force constant in the excited state. Especially in large molecules, the majority of the modes are silent, but the dephasing representation produces an artificially fast decay of fidelity amplitude [40], which in turn gives rise to artificially broadened spectra, often to the point that any structure is lost. Typical molecules are slightly anharmonic, so one cannot always use simple semiclassical methods such as the thawed Gaussian approximation [43], but they are not very chaotic, and hence the surprising accuracy of dephasing representation in chaotic systems does not help. Yet, the second-order approximation (3.27) for *f*(*t*), which is, by definition, exact in harmonic systems with different force constants, could—if evaluated efficiently—provide an accurate method for computing electronic molecular spectra even in the presence of anharmonicity and wavepacket splitting.

## 5. Conclusion

In conclusion, we derived a path integral formula for the quantum fidelity amplitude, which bypasses the conventional semiclassical approximations of past publications. Our first approach used a coordinate path integral for continuous systems and benefited from the explicit connection with the classical Liouville propagator. We note that this path integral approach allows in principle to incorporate the influence of the environment using the familiar Feynman–Vernon formalism. All that is required is adding the appropriate bath terms to the action. The effect of thermal noise would be to broaden the delta functions that arise from the 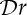 integration, leading to smearing of the phase factor in equation (2.20).

Our second approach relied on the phase-space path integral for kicked quantum maps. In the latter context, we also obtained an exact expansion of the exponent of the path integral and derived explicit expressions for the fidelity amplitude in the zeroth-, first- and second-order expansions; the first-order expansion yields exactly the dephasing representation, whereas the second-order expansion yields an approximation which corrects several drawbacks of the dephasing representation and other approximations based on linearizing the semiclassical propagator or path integral. It remains to be seen if it can be implemented efficiently.

Finally, the rigorous manipulation of the path integral has allowed us to make several rigorous statements about the validity of various approximations for fidelity amplitude.
